# Differential utilization of NF-kappaB RELA and RELB in response to extracellular versus intracellular polyIC stimulation in HT1080 cells

**DOI:** 10.1186/1471-2172-12-15

**Published:** 2011-02-10

**Authors:** James J Yun, Ming-Sound Tsao, Sandy D Der

**Affiliations:** 1 Department of Laboratory Medicine and Pathobiology, Faculty of Medicine, University of Toronto, Toronto, Ontario, M5S1A8 Canada; 2 Applied Molecular Oncology, Ontario Cancer Institute/University Health Network, Toronto, Ontario, M5G2M9 Canada

## Abstract

**Background:**

Pattern recognition receptors (PRRs) for double-stranded RNA (dsRNA) are components of innate immunity that recognize the presence of viral infection and initiate efficient defense mechanisms. In addition to previously well-characterized signaling pathways that are mediated by PKR and TLR3, new intracellular dsRNA sensors, that are members of CARD and DExD/H box helicase family, have been identified. However, the molecular mechanisms involved in the signaling pathways mediated by these new dsRNA sensors have not been extensively characterized.

**Results:**

Here, we studied an intracellular dsRNA pathway in the human fibrosarcoma cell line HT1080, which is distinct from the TLR3-mediated extracellular dsRNA pathway. Particularly, the NF-kB subunits RELA and RELB were differentially utilized by these two dsRNA signaling pathways. In TLR3-mediated dsRNA signaling, siRNA knock-down studies suggested a limited role for RELA on regulation of interferon beta and other cytokines whereas RELB appeared to have a negative regulatory role. By contrast, intracellular dsRNA signaling was dependent on RELA, but not RELB.

**Conclusions:**

Our study suggests that extracellular and intracellular dsRNA signaling pathways may utilize different NF-kB members, and particularly the differential utilization of RELB may be a key mechanism for powerful inductions of NF-kB regulated genes in the intracellular dsRNA signaling pathway.

## Background

Pattern recognition receptors (PRRs) are key players in host innate immune response against microbial pathogens. In order to launch effective defense mechanisms in response to viral infections, a number of cellular sensors that recognize universal components common to many viruses have been characterized. Double-stranded RNA (dsRNA) is one of the components that mammalian cells have developed several different receptors for since most viruses produce dsRNA during replication [1-3].

Interferon-inducible double-stranded RNA activated protein kinase (PKR) has long been studied as an intracellular sensor for viral dsRNA. PKR was initially characterized to participate in the mechanism that shuts down cellular translation to suppress viral replication and is now believed to be involved in a wide range of other cellular responses to viral infection [4]. Toll-like receptor 3 (TLR3) has been considered to be essential for mediating NF-kB-inducible gene responses to polyIC, a synthetic analogue of viral dsRNA [5], but there has yet been any strong evidence of physical interaction between TLR3 and viral dsRNA. The precise cellular location of TLR3 is still under discussion, but generally it is thought to be cell type dependent. TLR3 is expressed on the cell surface of fibroblast, but in two subtypes of dendritic cells it is thought to be located in endosomal compartments and transported to cell surface upon polyIC stimulation [6]. However, the role of TLR3 in innate immunity was soon questioned when TLR3 knock-out mice had no significant defect against virus challenges [7,8]. More recently, Retinoic acid inducible gene I (RIG-I) and Melanoma differentiation-associated gene 5 (MDA5), both RNA helicases, were reported to be novel and important intracellular regulators of polyIC-mediated signaling pathway leading to the activation of NF-kB [9,10]. Embryonic fibroblasts from RIG-I knock-out mice showed substantial defects in activation of NF-kB inducible genes participating in immune defense [11]. Subsequent studies have indicated cell type specific involvements of these dsRNA receptors [11,12].

Until the novel function of these RNA helicases, RIG-I and MDA5, was discovered, a dogma of dsRNA mediated signaling has been a separated or integrated signaling pathway between TLR3-dependent extracellular recognition of viral dsRNA and PKR-mediated intracellular recognition of viral dsRNA. Particularly in the TLR3-PKR integrated model [13], the recognition of viral dsRNA by TLR3 activates signaling cascades that include PKR, leading to the activation of NF-kB and Interferon regulatory factor 3 (IRF3). As a result, interferon genes are induced by synergy between NF-kB and IRF3, and other NF-kB-inducible inflammatory genes are also activated. PKR is believed to initiate a similar signaling pathway somewhere downstream because PKR could function as an internal receptor for dsRNA as well as a second messenger in TLR3 pathway. Therefore, whether these two pathways are indeed integrated or separated, TLR3-mediated signaling pathway has been considered to be a key route of anti-viral responses. However, according to the recent studies, RIG-I/MDA5-mediated signaling pathways seem to be not only TLR3-independent but also quite distinct from TLR3 pathway in terms of participating downstream molecules [9,10,14-18]. Although these findings imply that there are more than one dsRNA signaling pathway, the mechanism of action for additional intracellular (i.e. RIG-I/MDA5 dependent) pathways is not yet clear.

A number of signaling pathways in innate immunity eventually lead to the activation of NF-kB because its activation is critical for the induction of many key genes in host defense systems. So far, five members of NF-kB family have been identified: NF-kB1 (p50/p105), NF-kB2 (p52/p100), c-REL, RELA (p65) and RELB (I-REL). A functionally active NF-kB transcription factor consists of homodimers or heterodimers of NF-kB members. The p50/p65 heterodimer represents the proto-typical NF-kB factor although a number of different combinations of functional dimers are possible. The primary mechanism by which NF-kB activity is regulated involves pre-existing NF-kB dimers that are sequestered in cytoplasm and held inactive by inhibitor proteins such as Inhibitor of kB (IkB)-1 and -2 [19,20]. When appropriate signaling cascades are activated, phosphorylation of inhibitor molecules by upstream kinases leads to the dissociation and degradation of the inhibitors through a ubiquitination pathway, and the released and activated NF-kB dimers can then translocate into nucleus to function as transcriptional regulators [19,20]. Although NF-kB has been extensively studied, the differential role and utilization of each member is not fully understood due to its complex nature and the inherent variations in cell types and in signaling cascades used in different studies.

In this study, we show that there are two distinct dsRNA signaling pathways exist and that these pathways utilize different signaling molecules, particularly NF-kB RELA and RELB. We also propose that the utilization of RELB may be the key mechanism of powerful induction of IFNB and other inflammatory genes in response to intracellular dsRNA.

## Results

### Differential cellular responses to extracellular and intracellular polyIC treatments

The human fibrosarcoma cell line, HT1080, has been extensively used as a model for studying IFN signaling [21]. It is known to respond to dsRNA [22] and express functional TLR3 [23]. Given recent interest in intracellular dsRNA-sensing mechanisms, we studied the responses of HT1080 cells to extracellular stimulation by polyIC, via addition to the culture medium, as compared to intracellular stimulation, by transfection of the polyIC. Steady-state mRNA levels of IFNB, representing the prototypical response gene for dsRNA sensing, were measured using quantitative real-time PCR (qPCR). A robust induction of IFNB was observed at 40 μg/ml of extracellular polyIC (ex-polyIC), but interestingly HT1080 cells exhibited significantly greater sensitivity to intracellular stimulation by polyIC transfection as the dose response curve was shifted by more than two orders of magnitude (data not shown). Specifically, 0.4 μg/ml of intracellular polyIC (in-polyIC) treatment induced even greater IFNB steady-state mRNA expression levels than 40 μg/ml of ex-polyIC treatment. From this basic observation, we were interested to determine whether these two forms of dsRNA sensing were simply quantitatively different or qualitatively distinct in their mechanisms of regulation and biological functions.

**Figure 1:**
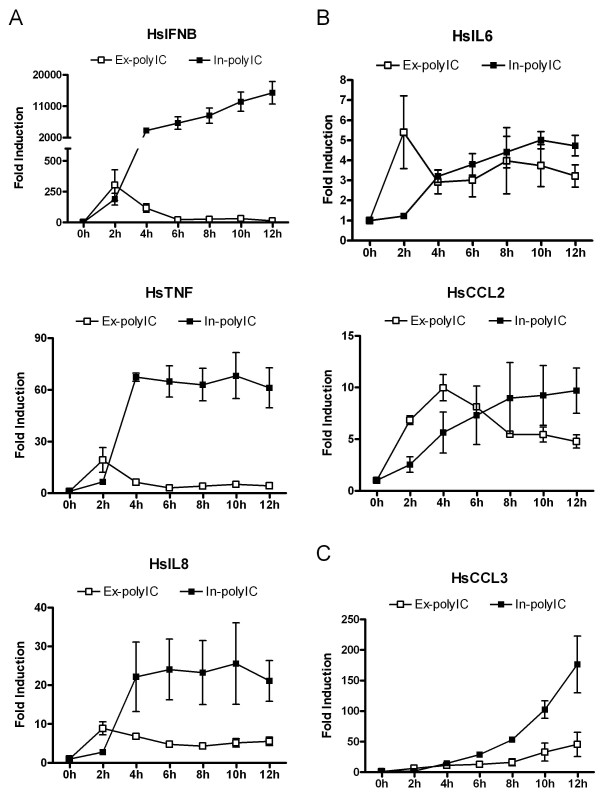
**Extracellular and intracellular polyIC treatments induce genes with different kinetic and magnitude**. HT1080 cell line was treated with 40 μg/ml ex-polyIC (open square) and 0.4 μg/ml in-polyIC (closed square) for 0 to 12 hours. Gene expression was measured using qPCR and the mean from triplicate experiments was calculated. Error bars represent standard error. A. IFNB, TNF and IL8 inductions in response to extracellular and intracellular polyIC treatments have both different kinetic and magnitude. B. IL6 and CCL2 inductions in response to extracellular and intracellular polyIC treatments have similar magnitude but different kinetic. C. CCL3 inductions in response to extracellular and intracellular polyIC treatments have similar kinetic but different magnitude.

We studied the kinetics of responses in HT1080 cells to extracellular vs. intracellular dsRNA for several known NF-kB-dependent genes (IL6, IL8, TNF, CCL2 and CCL3) along with IFNB. HT1080 cells were treated with 40 μg/ml ex-polyIC or 0.4 μg/ml in-polyIC for 0, 2, 4, 6, 8, 10 and 12 hours, and the mRNA levels of these genes were measured using qPCR. We observed three distinct expression patterns among these genes in response to ex-polyIC and in-polyIC. IFNB exemplified one pattern in which ex-polyIC resulted in a short phase of elevated mRNA accumulation, peaking at 2 hours (Figure 1A) followed by a well-documented down-regulation phase [24], whereas in-polyIC led to both a substantively increased magnitude of induction and an extended period of elevated mRNA steady-state levels, for at least up to 12 hours (Figure 1A). This pattern was similarly observed for TNF and IL-8 (Figure 1A). Alternately, a second group of genes, exemplified by IL-6 and CCL2 (Figure 1B), exhibited similar kinetics to the first pattern in response to intracellular and extracellular polyIC stimulations with a moderate (~5-10 fold) and sustained induction over 12 hours. CCL3 expression represented a third pattern with similar kinetics of mRNA level inductions between ex-polyIC and in-polyIC stimulation, that was slow and progressive, but in which in-polyIC provided a higher magnitude of accumulation than ex-polyIC (Figure 1C).

**Figure 2:**
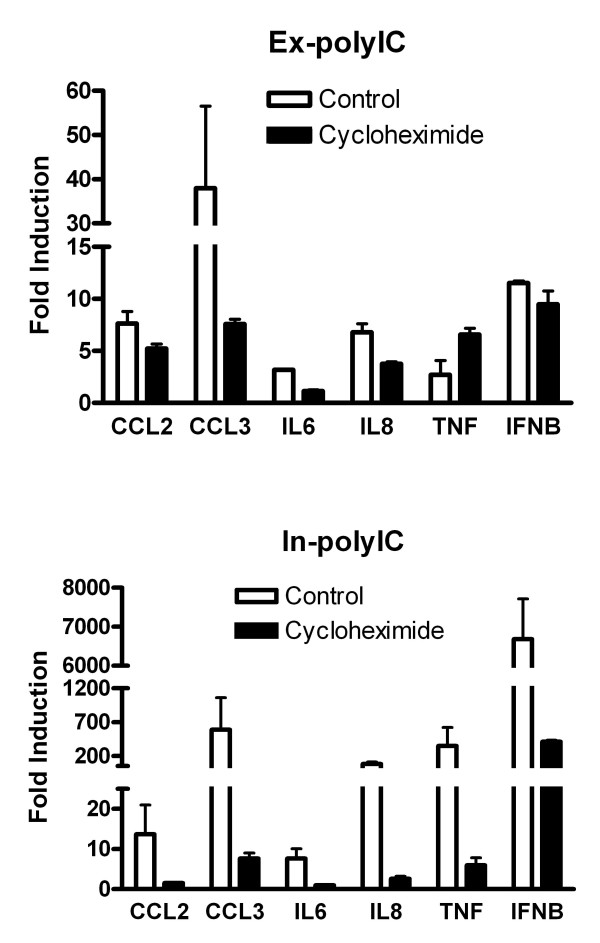
**Blocking protein synthesis diminishes substantial gene inductions by in-polyIC stimulation**. HT1080 cell line was treated with 40 μg/ml ex-polyIC and 0.4 μg/ml in-polyIC for 8 hours in the presence/absence of 10 μg/ml cycloheximide. Gene expression was measured using qPCR and the mean from triplicate experiments was calculated. Error bars represent standard error. Gene inductions in response to ex-polyIC are not significantly affected by cycloheximide treatment whereas gene inductions in response to in-polyIC are diminished by cycloheximide treatment.

The substantive magnitude and extended period of gene inductions by polyIC has been shown to involve positive feedback loops. While it is known that autocrine IFN production provides a positive feedback loop for ex-polyIC and some viruses, it is yet unclear whether autocrine IFNs may play a similar role for in-polyIC. Unless negative feedback mechanisms are activated, the cellular signals initiated by these genes can be amplified. Therefore, we studied the effect of cycloheximide, which blocks new protein synthesis, on mRNA level inductions in response to ex-polyIC and in-polyIC stimulation. HT1080 cells were treated with 40 μg/ml ex-polyIC or 0.4 μg/ml in-polyIC for 8 hours in the absence/presence of 10 μg/ml cycloheximide. The substantive induction of these genes by in-polyIC was diminished when new protein synthesis was blocked whereas gene inductions in response to ex-polyIC stimulation were relatively not inhibited (Figure 2). These findings suggest that distinct regulatory mechanisms govern intracellular vs. extracellular dsRNA sensing in HT1080 cells.

### Differential induction of anti-viral activity by extracellular and intracellular polyIC

**Figure 3:**
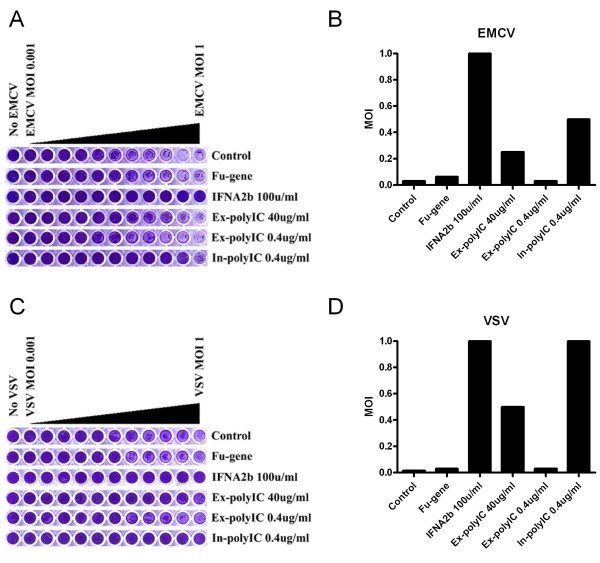
**In-polyIC treatment induces effective antiviral protection**. HT1080 cells were challenged with virus after various pre-treatments including extracellular and intracellular polyIC for 7 hours. IFNA2A treatment was a positive control to show that biologically significant interferon pre-treatment protects cells against virus. Fu-gene (transfection reagent) and 0.4 μg/ml of ex-polyIC were negative controls to show neither of them alone elicits anti-viral protection. Cell viability was measured with crystal violet staining that shows live cells in purple. A. HT1080 cells were challenged with a serial dilution (from MOI 1 to 0.001) of encephalomyocarditis virus (EMCV). B. Summary of Figure 3A. Amount of input EMCV virus (MOI) that causes 50% (or greater) cell survival across different polyIC treatments. C. HT1080 cells were challenged with a serial dilution (from MOI 1 to 0.001) of vesicular stomatitis virus (VSV). D. Summary of Figure 3C. Amount of input VSV virus (MOI) that causes 50% (or greater) cell survival across different polyIC treatments.

We were particularly interested in the substantive induction of IFNB in response to a relatively small input of in-polyIC. To examine the biological significance of this response, we assessed the antiviral activity induced by in-polyIC as compared to ex-polyIC treatment (Figure 3). HT1080 cells were pre-treated with ex-polyIC or in-polyIC for 7 hours and then challenged with encephalomyocarditis virus (EMCV) for 30 hours or vesicular stomatitis virus (VSV) for 40 hours with a wide range of Multiplicity of Infections (MOIs). Under these conditions, the observed median Tissue Culture Infectious Dose (TCID_50_) for EMCV and VSV was approximately MOI = 0.01-0.03. Whereas extracellular stimulation with 0.4 μg/ml polyIC was essentially ineffective, transfection with the same amount of polyIC provided near complete protection against viral challenge extending up to MOI = 1. This represented a comparable level of antiviral protection to that provided by treatment with 100 U/ml of recombinant IFN-alpha. Curiously, while 40 μg/ml ex-polyIC was ineffective in conferring protection against EMCV, some antiviral activity against VSV was observed although still less than that induced by in-polyIC 0.4 μg/ml. These results indicate that the substantive stimulation of IFNB expression by in-polyIC is associated with significant antiviral protection although the precise proportions which are IFNB-dependent or -independent is still unclear.

### Downstream mediators of extracellular and intracellular polyIC signaling pathways

**Figure 4:**
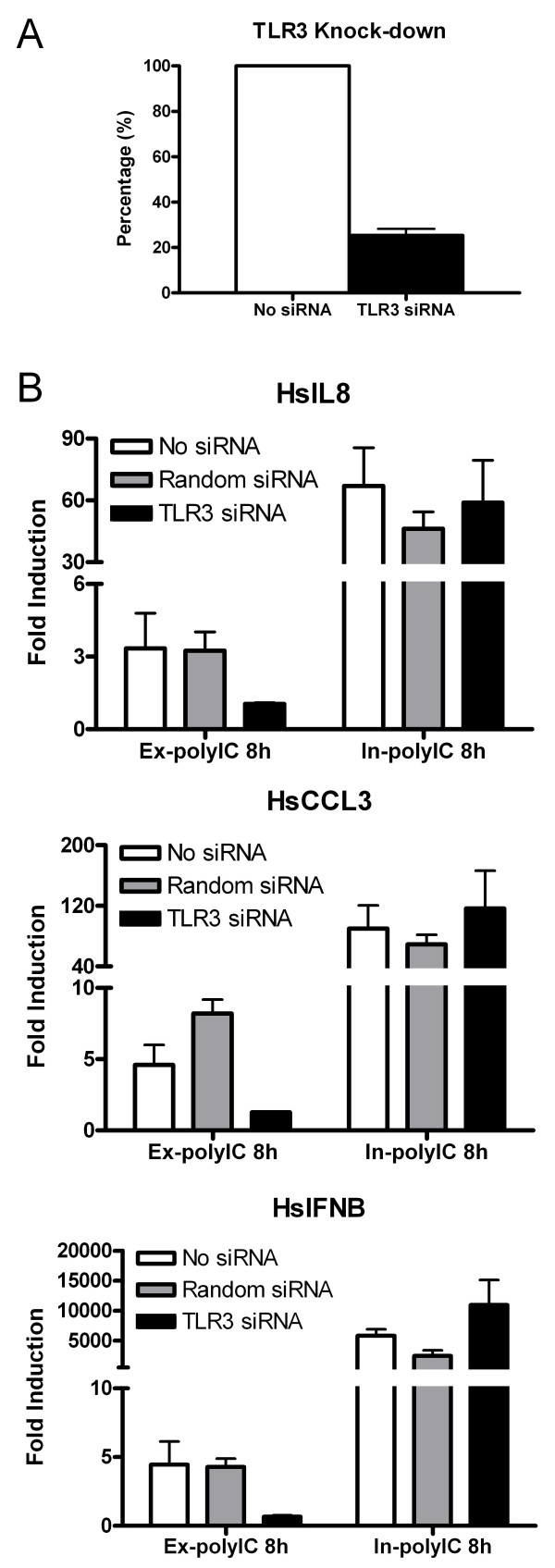
**Involvement of TLR3 in gene activation in response to extracellular and intracellular polyIC treatments**. HT1080 cell line was treated with 40 μg/ml of ex-polyIC and 0.4 μg/ml of in-polyIC for 8 hours after knocking down TLR3 using gene-specific siRNA. Control siRNA with random siRNA sequences was included to monitor non-specific inhibition. Gene expression was measured using qPCR and the mean from triplicate experiments was calculated. Error bars represent standard error. A. Nucleofection of TLR3 siRNA achieved about 75% knock-down efficiency. B. IL8, CCL3 and IFNB inductions in response to ex-polyIC treatment are dependent on TLR3 whereas the same gene inductions in response to in-polyIC treatment are independent of TLR3.

TLR3 represents the prototypical extracellular dsRNA sensor, although its intracellular localization in specific cell types has also been reported [6,25]. While functional TLR3 in HT1080 cells has been reported [23], we verified its function as a dsRNA sensor in this cell line using TLR3 specific siRNA. Knock-down efficiency of ~75% was achieved for TLR3 using siRNA nucleofection (Figure 4A). The induction of most genes (IFNB, IL6, IL8, TNF, CCL2 and CCL3) in response to ex-polyIC (40 μg/ml for 8 h) was decreased when TLR3 was knocked down whereas the induction of the same genes in response to in-polyIC (0.4 μg/ml for 8 h) was not significantly affected, represented by IL8, CCL3 and IFNB (Figure 4B). Similar experiments with 2 and 6 hour treatments showed similar results (data not shown).

We also took advantage of the human embryonic kidney 293 (HEK293) cell line, which is known to lack functional TLR3 expression [5], in order to examine intracellular dsRNA signaling. As expected, no response for IFNB was observed when HEK293 cells were stimulated with up to 40 μg/ml ex-polyIC but 15-fold induction of IFNB was detected following transfection with 0.4 μg/ml in-polyIC (supplemental data 1).

### Involvement of IKK-2 in extracellular and intracellular polyIC signaling pathways

**Figure 5:**
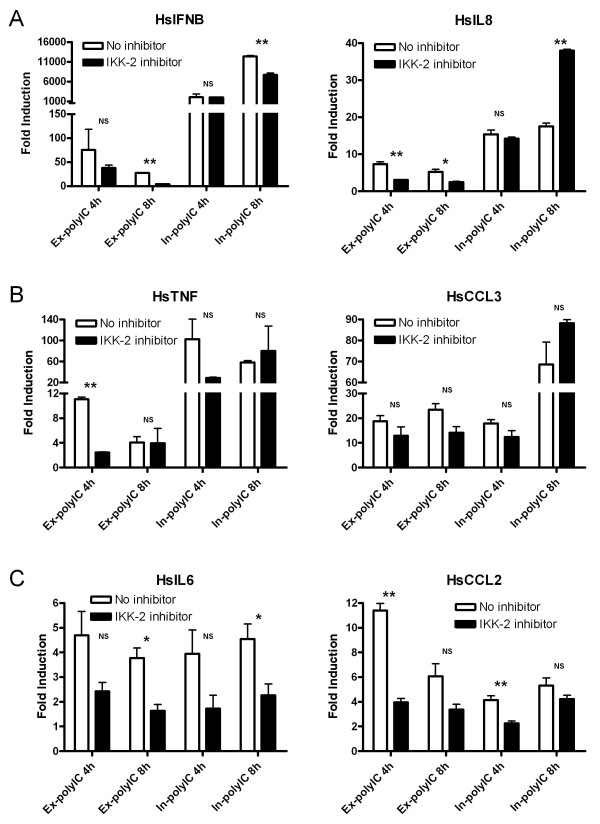
**Involvement of IKK-2 in gene activation in response to extracellular and intracellular polyIC treatments**. HT1080 cell line was treated with 40 μg/ml of ex-polyIC and 0.4 μg/ml of in-polyIC for 4 and 8 hours in the absence/presence of IKK-2 inhibitor. Gene expression was measured using qPCR. Graphs show the average of two or three independent experiments, and the student's t-test was performed to indicate statistically significant differences between untreated control and IKK-2 inhibitor-treated cells. (* is for P-value≤0.05, ** is for P-value≤0.01 and NS is 'not significant'.) A. IFNB and IL8 inductions in response to in-polyIC treatment are not significantly affected by IKK-2 inhibition whereas the same gene inductions in response to ex-polyIC treatment are affected by IKK-2 inhibition. B. TNF and CCL3 inductions in response to both ex-polyIC and in-polyIC are relatively not affected by IKK-2 inhibition. C. IL6 and CCL2 inductions in response to both ex-polyIC and in-polyIC are affected by IKK-2 inhibition.

IKK-2 is believed to play a central role in activating NF-kB as a result of TLR3 activation [3]. Since the in-polyIC signaling pathway seems to be TLR3 independent, the involvement of IKK-2 was studied in HT1080 cells using an inhibitor, IKK-2 inhibitor IV (Calbiochem). IFNB, IL6, IL8, TNF, CCL2 and CCL3 mRNA levels were measured in response to ex-polyIC (40 μg/ml for 4 h and 8 h) or in-polyIC (0.4 μg/ml for 4 h and 8 h) in the presence of 5 μM IKK-2 inhibitor IV. Induction of IFNB and IL8 by ex-poly IC was diminished by IKK-2 inhibitor IV, but the induction of these genes by in-polyIC was not affected (Figure 5A). TNF and CCL3 inductions by ex-polyIC and in-polyIC were relatively insensitive to IKK-2 inhibitor IV (Figure 5B) although IL6 and CCL2 inductions by both types of polyIC stimulation were diminished by this inhibitor (Figure 5C).

### Differential roles of RELA and RELB in gene expression in response to extracellular and intracellular polyIC treatments

**Figure 6:**
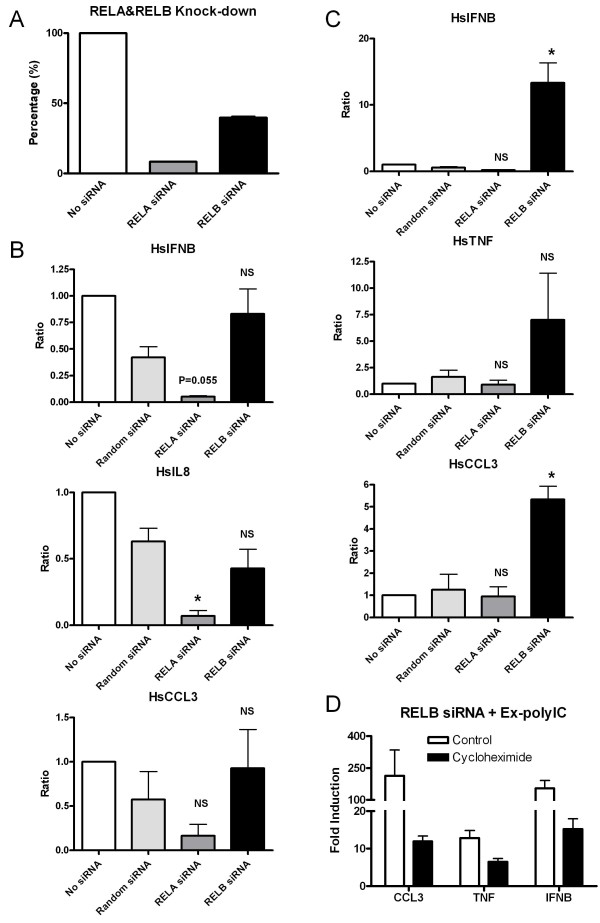
**Involvement of RELA and RELB in gene activation in response to extracellular and intracellular polyIC treatments**. HT1080 cell line was treated with 40 μg/ml of ex-polyIC and 0.4 μg/ml of in-polyIC for 8 hours after knocking down RELA and RELB using gene-specific siRNAs. Gene expression was measured using qPCR. Graphs show the average of two independent experiments, and the student's t-test was performed to indicate statistically significant differences between non-specific siRNA control and gene-specific knock-down cells. (* is for P-value≤0.05, ** is for P-value≤0.01 and NS is 'not significant'.) A. Nucleofection of RELA and RELB siRNAs achieved about 92% and 60% knock-down efficiencies, respectively. B. IFNB, IL8 and CCL3 inductions in response to in-polyIC treatments are dependent on RELA but independent on RELB. C. IFNB, TNF and CCL3 inductions in response to ex-polyIC treatment are independent of RELA but dependent on RELB. Particularly, IFNB, TNF and CCL3 inductions in response to ex-polyIC treatment are significantly increased in RELB knock-down. D. HT1080 cell line with RELB-knocked-down was treated with 40 μg/ml of ex-polyIC in the absence/presence of 10 μg/ml cycloheximide. The increase in gene inductions of IFNB, TNF and CCL3 is diminished by cycloheximide treatment.

As our results suggested that in-polyIC and ex-polyIC signaling pathways differ in several aspects, we next assessed whether these two pathways utilize different NF-kB members for mediating downstream transcription regulation. Using siRNA targeting RELA and RELB, knock-down efficiency of ~92% and ~60%, respectively, was achieved in HT1080 cells (Figure 6A). Under these conditions, the induction of IFNB, IL8 and CCL3 in response to in-polyIC (0.4 μg/ml for 8 h) was diminished by RELA knock-down but not affected by RELB knock-down (Figure 6B). Interestingly, while ex-polyIC stimulation (40 μg/ml for 8 h) was unaffected by RELA knock-down, the induction of IFNB, TNF and CCL3 was significantly increased by RELB knock-down (Figure 6C). This experiment was repeated using a second set of RELA and RELB siRNAs and yielded similar results (supplemental data 2).

RELB may have an inhibitory role on gene inductions by ex-polyIC stimulation, while the same inhibitory role may not be seen in in-polyIC signaling. As a result, the increase in gene induction by ex-polyIC in the absence of RELB may also work through the same positive feedback mechanism that induced high level of mRNA levels in in-polyIC signaling. To test this hypothesis, HT1080 cells with RELB knock-down were treated with ex-polyIC (40 μg/ml for 8 h) in the absence/presence of 10 μg/ml cycloheximide. Interestingly, the increase in gene inductions by ex-polyIC previously seen in RELB knock-down was diminished (Figure 6D). These results suggest that two types of polyIC stimulation induce gene expression through differential usage of NF-kB members RELA and RELB.

## Discussion

Recent studies have established there are distinct dsRNA signaling pathways in innate immunity although the new intracellular pathways, involving two or possibly more members of CARD and DExD/H box helicase family, are not yet extensively mapped out. We have observed that the gene expression patterns induced by the extracellular and intracellular polyIC are different in HT1080 fibroblast cells, suggesting that at least two distinct dsRNA signaling pathways may exist (Figure 1). We also showed that the in-polyIC response is TLR3-independent (Figure 4), in concordance with the recent studies.

Our study validated the observations of previous studies but also revealed novel aspects of the intracellular dsRNA pathway. First, the substantive induction of genes by in-polyIC stimulation was diminished when protein synthesis was blocked using cycloheximide (Figure 2). We will discuss this finding further in the context of the inhibitory role of RELB in NF-kB signaling pathways. Secondly, the substantial induction of IFNB by in-polyIC was shown to have biological significance demonstrating effective protection against virus challenges (Figure 3). Thirdly, our results showed that IKK-2 is critical for the expression of NF-kB inducible genes in both extracellular and intracellular dsRNA pathways. However, it was also indicated that some NF-kB inducible genes may be activated through IKK-2-independent mechanism in response to in-polyIC (Figure 5).

Differential utilization of NF-kB members in a number of different biological systems has been reported. Several groups have studied the differential usage of NF-kB RELA and RELB. Earlier studies have observed that the activation of RELA is inducible whereas RELB is constitutively activated in mouse lymphoid tissues [26-29]. These studies as well as others have shown that RELB is not inhibited by IkB-1 or IkB-2 [26,29-31]. Furthermore, subsequent studies using mouse embryonic fibroblast (MEF) have shown that the activation of RELB is not through a classical IKK complex containing IKK-2 and NEMO, but rather through IKK-1 and NIK in several signaling pathways mediated by lymphotoxinB [32-35], EBV latent membrane protein 1 [36,37], and RSV [38]. These distinct activation mechanisms are closely linked to the difference in activation kinetics between RELA and RELB. Generally, the activation of RELA was shown to be fast and transient whereas the activation of RELB tended to be gradual but long-lasting [33,39-42]. Although our study suggests there are both IKK-dependent and -independent mechanisms that mediate responses to different forms of polyIC stimulation, the specific molecular mechanisms involved remains to be understood. Specifically, some possibilities include differential activation of the IKK complex or specific regulation of RelA in response to in-polyIC stimulation.

Several mechanisms of regulation regarding RELA and RELB have been described. The transcription of RELB could be induced by RELA activation [39], indicating that the activated RELA may contribute to the latent but enhanced activation of RELB. This fits into the observation of exchanging dimers, in which the activation of RELA is quickly down-regulated by the induction of IkBs but RELB switches with RELA in gene promoter regions for the prolonged activation of target genes [41]. Furthermore, even in the absence of functional RELA, it is shown that RELB may compensate for the loss of RELA in development [43]. On the other hand, RELB may possess a regulatory effect on the expression of inflammatory genes. RELB knock-out mice was reported to generally suffer multi-organ inflammation [19,20,44,45], and MEF from these mice showed a persistent induction of several chemokines [46]. Interestingly, RELB was shown to form an inactive dimer with RELA in TNF-treated MEF [47,48]. This phenomenon was initially understood as RELB inhibiting activated RELA [48] but later explained that RELA inhibits RELB to block prolonged RELB-mediated gene transcription [47].

Our results indicated that RELA may be the major transcription factor inducing the group of NF-kB regulated genes we tested in both intracellular and extracellular dsRNA signaling pathways. Our preliminary data showed that ex-polyIC induced nuclear translocation of RELA at early hours (2 and 4 hour) whereas in-polyIC induced nuclear translocation of RELA at late hours (8, 10 and 12 hour) (unpublished data). Interestingly, in the ex-polyIC signaling pathway, our results suggested a negative regulatory role for RELB had on the induction of RELA-dependent inflammatory genes (Figure 6). Specifically, although knock-down of RELB alone was insufficient for induction of these inflammatory genes, it provided enhanced expression following ex-polyIC stimulation. We had considered the possibility that the apparent lack of RELB involvement in in-polyIC signaling may have been due to inefficient knock-down (~60% for RELB, Figure 6A), but the unexpected enhancement of ex-polyIC induction of downstream genes suggests in fact that the RELB knock-down did have some effect on cellular responses to dsRNA. Conversely, as non-specific or off-target effects of siRNA treatments are well-known, these mechanisms may have accounted for the enhancement of extracellular responsiveness to ex-polyIC by RELB knock-down. However, the concomitant lack of enhanced responsiveness to extracellular signaling in other gene-specific-siRNA-treated cells and to intracellular signaling in the same RELB-siRNA-treated cells argues against such non-specific effects. Lastly, the similar results on differential responses to extracellular and intracellular polyIC provided by a second distinct RELB-siRNA further supports the specificity of this observation.

**Figure 7:**
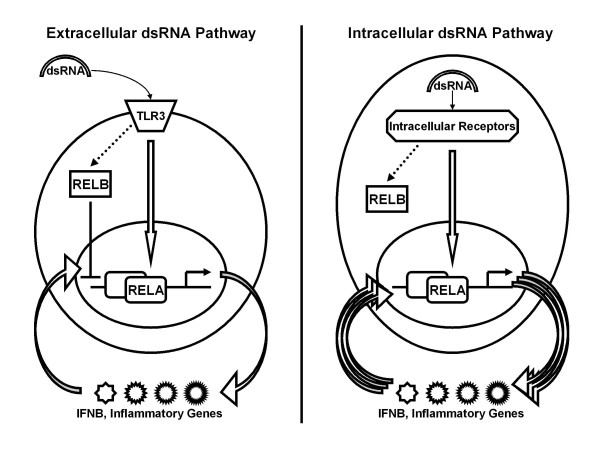
**Proposed mechanism of gene regulation in response to extracellular and intracellular dsRNA stimulation**. TLR3-mediated extracellular dsRNA signaling pathway activates IFNB and inflammatory genes through NF-kB RELA but the subsequent activation of RELA by secondary signals is inhibited by RELB (left panel). On the other hand, secondary signals induced by the activation of NF-kB RELA in the intracellular dsRNA signaling pathway are not inhibited by RELB resulting in a positive feedback loop of RELA activation leading to powerful activation of IFNB and inflammatory genes (right panel).

The mechanistic role of RELB in the intracellular dsRNA signaling is not clear though the translocation of RELB to nucleus in response to in-polyIC was observed in HT1080 cells (unpublished data). RELB seems to neither drive the powerful induction of IFNB and other inflammatory genes nor to inhibit RELA-mediated gene transcription in response to in-polyIC (Figure 6). It would appear that in HT1080 fibroblasts, RELB primarily functions as a negative regulator of transcriptional activation in response to extracellular dsRNA sensing. Given its known participation in forming heterodimers with RELA, some form of squelching may be responsible for such a negative regulatory role. However, non-transcriptional mechanisms cannot be ruled out. For example, RELB may be required for regulating the expression of a protein that controls mRNA stability. As genes like IFNB that contain AU-rich elements in their 3' UTR are known to be actively targeted for mRNA degradation, the loss of such a function could account for the enhanced steady-state IFNB levels we observed in cells with knock-down of RELB. Whether transcriptional or non-transcriptional, the essential role of inhibitory RELB may be to interfere early with the positive feedback loop created by dsRNA-inducible genes that are both NF-kB-regulated and NF-kB-activating. Figure 7 illustrates this proposal. In ex-polyIC signaling the inhibitory RELB may cut off the loop early contributing to the down-regulation phase, which was observed in many genes (Figure 1), whereas in in-polyIC signaling or in RELB-knocked-down ex-polyIC signaling the positive feedback loop may not be interfered with RELB but can be diminished by blocking protein synthesis. We would emphasize that this proposed mechanism may only be relevant to fibroblasts because other types of cells participating in innate immune response (such as macrophages and dendritic cells) may utilize these pattern recognition receptors in different manners. In other words, RELB may not have any inhibitory role in RELA-mediated gene responses in these other cell types.

Lastly, other molecules involved in dsRNA signaling, including IRF3 and MAPK have been observed to exhibit different patterns of activation in response to these two different types of polyIC stimulation [49]. Therefore, consistent with our observations of differential activation of RELA and RELB, the theme of differential activation of transcription factors by different forms of polyIC stimulation seems generalizable. It would be important to study how these additional factors may interact with RELA and RELB for more complete understanding of gene regulation in response to extracellular and intracellular polyIC treatments.

## Conclusions

Our study shows that the two general categories of extracellular vs. intracellular dsRNA signaling pathways may utilize different NF-kB members, and particularly the differential utilization of RELB may be a key mechanism to drive powerful inductions of NF-kB regulated genes in the intracellular dsRNA signaling pathway.

## Methods

### Cell culture, reagents and polyIC treatment

HT1080 human fibrosarcoma cell line was cultured in DMEM with 10% fetal bovine serum. IKK-2 inhibitor IV was added to culture medium at 5 μM concentration (Calbiochem, EMD Biosciences Inc). Transfection of polyIC (Amersham Biosciences/GE Healthcare) was performed with FuGENE 6 transfection reagent (Roche Applied Science) according to the manufacturer's protocol. Cycloheximide (C4859) was purchased from Sigma-Aldrich Co.

### Reverse transcription (RT) reaction and quantitative real-time PCR (qPCR)

RNA extraction was performed according to the manufacturer's protocol for the RNeasy Kit (QIAGEN). DNase treatment was performed on RNA samples using DNA-*free *(Ambion Inc.) according to the manufacturer's protocol in order to remove genomic DNA contamination. For reverse transcription to generate cDNA, 5 μg of RNA was mixed with RNase-free water (QIAGEN) and 1 μl of oligo-dT_23 _(Sigma-Aldrich Co.) to a final volume of 12 μl, then incubated at 90°C for 10 minutes. To this, 4 μl of 5× First-Strand buffer, 2 μl of 0.1 M DTT, 1 μl of 10 mM dNTP, and 1 μl (200 units) of Superscript II (Invitrogen Co.) were added. The mixture was incubated at 42°C for 90 minutes followed by 15 minutes at 70°C.

qPCR reactions were performed using the ABI Prism 7900HT (PE Applied Biosystem, Perkin Elmer, Foster City, CA) in 384 micro-well plates. All samples, including the external standards and non-template control, were run in triplicate. The use of external standard and qPCR condition were previously described [50]. qPCR was monitored and analyzed by the Sequence Detection System ver. 2.0 (PE Applied Biosystem, Perkin Elmer, Foster City, CA). All qPCR primers used in this study are listed in supplemental data 3.

### Antiviral assay

All antiviral assays were performed in 96-well plate. 50,000 cells were plated in each well containing 100 μl of medium and incubated at 37°C overnight before any treatment. After each treatment, cells were challenged with EMCV (for 30 hours) or VSV (for 40 hours) at 37°C before staining. Live cells were stained with 0.5% crystal violet.

### siRNA knock-down

All siRNAs (Control - 1022076, TLR3 - SI00050043, DDX58 - SI00361809, IFIH1 - SI03648981, RELA - SI00131943 and SI00301672, and RELB - SI00089117 and SI00089131) were purchased from QIAGEN, and nucleofection was performed using AMAXA Biosystems according to the manufacturer's protocol specified as optimal for HT1080 (Solution V with nucleofection mode A-23). The efficiency of each gene-specific siRNA knock-down was calculated based on mRNA levels measured by quantitative real-time PCR whereby the percentage represents the resulting mRNA level of each gene knockdown compared to corresponding control.

## Authors' contributions

JJY conceived and coordinated the project, performed all experiments, constructed all figures and tables, and wrote the manuscript. MST supervised the project and helped writing the manuscript. SDD conceived and supervised the project, and wrote the manuscript. All authors read and approved the final manuscript.

## Supplementary Material

Additional file 1 **293 cell line treated with ex-polyIC (40 μg/ml) and in-polyIC (0.4 μg/ml) for 8 h**. Click here for file

Additional file 2 **The involvement of RELA and RELB in gene activation in response to extracellular and intracellular polyIC treatments. (Second set of siRNAs)**. Click here for file

Additional file 3 **Real-time PCR primers**. Click here for file
