# Supplementary material for: Differential utilization of NF-kappaB RELA and RELB in response to extracellular versus intracellular polyIC stimulation in HT1080 cells
Source: BMC Immunol. 2011 Feb 10;12:15. doi: 10.1186/1471-2172-12-15 (PMC3048558; doi:10.1186/1471-2172-12-15)
Supplement: Additional file 1 — 293 cell line treated with ex-polyIC (40 μg/ml) and in-polyIC (0.4 μg/ml) for 8 h. [file 1471-2172-12-15-S1.DOC]

Supplemental Data 1

293 cell line treated with ex-polyIC (40 μg/ml) and in-polyIC (0.4 μg/ml) for 8h
