# Supplementary material for: Differential utilization of NF-kappaB RELA and RELB in response to extracellular versus intracellular polyIC stimulation in HT1080 cells
Source: BMC Immunol. 2011 Feb 10;12:15. doi: 10.1186/1471-2172-12-15 (PMC3048558; doi:10.1186/1471-2172-12-15)
Supplement: Additional file 2 — The involvement of RELA and RELB in gene activation in response to extracellular and intracellular polyIC treatments. (Second set of siRNAs). [file 1471-2172-12-15-S2.DOC]

Supplemental Data 2

The involvement of RELA and RELB in gene activation in response to extracellular and intracellular polyIC treatments (Second set of siRNAs)
