# Supplementary material for: Differential utilization of NF-kappaB RELA and RELB in response to extracellular versus intracellular polyIC stimulation in HT1080 cells
Source: BMC Immunol. 2011 Feb 10;12:15. doi: 10.1186/1471-2172-12-15 (PMC3048558; doi:10.1186/1471-2172-12-15)
Supplement: Additional file 3 — Real-time PCR primers. [file 1471-2172-12-15-S3.DOC]

Supplemental Data 3

Real-time PCR primers

| Primer pair | Forward | Reverse |
| --- | --- | --- |
| HsB2M | GAGTGCTGTCTCCATGTTTGATGT | AAGTTGCCAGCCCTCCTAGAG |
| HsCCL2 | GGACCACCTGGACAAGCAAA | AGATTCTTGGGTTGTGGAGTGAGT |
| HsCCL3 | CCTCCCCCTTCCCTCACA | TGGTGCCATGACTGCCTACA |
| HsDDX58(RIG-I) | GACCACATCCCAAGCCAAAG | TCACATGGATTCCCCAGTCAT |
| HsIFNB1 | AGCAGTCTGCACCTGAAAAGATATT | TGTACTCCTTGGCCTTCAGGTAA |
| HsIL6 | CACTGGGCACAGAACTTATGTTG | AAAATAATTAAAATAGTGTCCTAACGCTCAT |
| HsIL8 | AATCTGGCAACCCTAGTCTGCTA | AAACCAAGGCACAGTGGAACA |
| HsRELA | TGCTTGGCAACAGCACAGA | AGCTGCTGAAACTCGGAGTTGT |
| HsRELB | TGGAGGAGCCGTGCAATC | AGGCTGAATATGTCCACTTCAGAAG |
| HsTLR3 | GGTCCCAAGCCTTCAACGA | GTGAAGGAGAGCTATCCACATTTTTA |
| HsTNF | GCCAGAATGCTGCAGGACTT | GGCCTAAGGTCCACTTGTGTCA |
